# Bioremediation of Explosive TNT by *Trichoderma viride*

**DOI:** 10.3390/molecules25061393

**Published:** 2020-03-19

**Authors:** Zeid A. Alothman, Ali H. Bahkali, Abdallah M. Elgorban, Mohammed S. Al-Otaibi, Ayman A. Ghfar, Sami A. Gabr, Saikh M. Wabaidur, Mohamed A. Habila, Ahmed Yacine Badjah Hadj Ahmed

**Affiliations:** 1 Chemistry Department, College of Science, King Saud University, Riyadh, Kingdom of Saudi Arabia, P.O. Box 2455, Riyadh 11451, Saudi Arabia; aghafr@ksu.edu.sa (A.A.G.); swabaidur@ksu.edu.sa (S.M.W.); mhabila@ksu.edu.sa (M.A.H.); ybadjah@ksu.edu.sa (A.Y.B.H.A.); 2 Botany and Microbiology Department, College of Science, King Saud University, P.O. Box 2455, Riyadh 11451, Saudi Arabia; abahkali@ksu.edu.sa (A.H.B.); motaibi@yahoo.com (M.S.A.-O.); 3 College of Applied Medical Sciences, King Saud University, Riyadh 11451, Saudi Arabia; dr.samigabr@gmail.com

**Keywords:** bioremediation, explosives, *Trichoderma viride*, GC/MS, microbial screening

## Abstract

Nitroaromatic and nitroamine compounds such as 2,4,6-trinitrotoluene (TNT) are teratogenic, cytotoxic, and may cause cellular mutations in humans, animals, plants, and microorganisms. Microbial-based bioremediation technologies have been shown to offer several advantages against the cellular toxicity of nitro-organic compounds. Thus, the current study was designed to evaluate the ability of *Trichoderma viride* to degrade nitrogenous explosives, such as TNT, by microbiological assay and Gas chromatography–mass spectrometry (GC–MS) analysis. In this study, *T. viride* fungus was shown to have the ability to decompose, and TNT explosives were used at doses of 50 and 100 ppm on the respective growth media as a nitrogenous source needed for normal growth. The GC/MS analysis confirmed the biodegradable efficiency of TNT, whereas the initial retention peak of the TNT compounds disappeared, and another two peaks appeared at the retention times of 9.31 and 13.14 min. Mass spectrum analysis identified 5-(hydroxymethyl)-2-furancarboxaldehyde with the molecular formula C_6_H_6_O_3_ and a molecular weight of 126 g·mol^−1^ as the major compound, and 4-propyl benzaldehyde with a formula of C_10_H_12_O and a molecular weight of 148 g mol^−1^ as the minor compound, both resulting from the biodegradation of TNT by *T. viride*. In conclusion, *T. viride* could be used in microbial-based bioremediation technologies as a biological agent to eradicate the toxicity of the TNT explosive. In addition, future molecular-based studies should be conducted to clearly identify the enzymes and the corresponding genes that give *T. viride* the ability to degrade and remediate TNT explosives. This could help in the eradication of soils contaminated with explosives or other toxic biohazards.

## 1. Introduction

Explosives are reactive chemical substances present in compounds or mixtures that contain a great amount of potential energy that can produce an explosion if released suddenly. This is usually accompanied by the production of light, heat, sound, and pressure. Nitro-aromatic and nitramine compounds, such as 2,4,6-trinitrotoluene (TNT), hexahydro-1,3,5-trinitro-1,3,5-triazine (RDX), and octahydrol-1,3,5,7-tetranitro-1,3,5,7-tetrazocine (HMX), are common military explosives that are found in soils at destruction ranges, explosive dumping grounds, industrial production sites, firing ranges, and ammunition factories [1,2,3]. The United States Environmental Protection Agency (USEPA) has listed Nitro-substituted explosives, including TNT and RDX, as priority pollutants, whereas RDX, classified as a potential carcinogen that is toxic to organisms, is comparatively mobile in the soil, has a low rate of degradation in soil, and presents distinct problems for bioremediation. Environmental pollution comes from nitrite industrial chemicals associated with vehicles, such as nitro explosives, dyes and polyurethane compounds, herbicides, pesticides, solvents, and others [4,5,6,7].

Many studies have discussed the synthesis of nitro-aromatic explosives such as 2,4,6-trinitrotoluene (TNT) and 2,4,6-trinitrophenol (picric acid) and their uses for military purposes because of their highly explosive properties, thermal stability, and their insensitivity to shock and friction [8,9]. In civilian industries, these compounds are used as raw materials for the manufacturing of pesticides, herbicides, pharmaceutical products, dyes, and explosives [10,11]. Thus, the extensive use of these explosives in military applications requires the implementation of extensive handling and disposal techniques, whereas their transformation products lead to increased environmental pollution—particularly in the soil, sediment, surface, and groundwater—to levels that threaten human health and the environment [12,13,14,15,16]. In addition, animal experimental studies have reported that the transformation products of TNT are teratogenic, cytotoxic, and may cause cell mutation; however, the carcinogenic effects of TNT on humans still need to be explored [17,18]. This may be related to the eco-toxicological effects and persistence of TNT and its transformation products in the environment, which significantly affects a wide range of ecological receptors, particularly microorganisms, algae, plants, invertebrates, some vertebrates, and humans [19,20,21]. Although several physico-chemical [8,22] and bioremediation technologies [23,24,25,26] have been applied to remediate environments polluted by TNT, only microbial-based bioremediation technologies offer a number of advantages [16,27]. It was reported that microbial-based bioremediation significantly uses the in situ microbial community to remediate toxic contaminates and return the polluted environment to its original state or at least minimize the toxicity of the environment toward the normal range [28,29,30,31,32].

Previous studies showed that many fungal species had the ability to decompose xenobiotic alien vehicles, including nitrogenous explosives, via transformation mechanisms using certain cellular degrading enzymes [30,31,32]. In addition, several studies have previously reported the ability of bacteria and fungi to neutralize and sustain the effective transformation or degradation of nitroaromatic pollutants [33,34]. Thus, fungi could be helpful as a biological control in the treatment of TNT and Composition C4 explosives [35,36,37,38]. Basidiomycetes—lignin-decomposing organisms such as *Phanerochaete chrysosporium*—have been shown to be the only organisms capable of mineralizing TNT [39,40].

Fungi of different species and habitats have been the subject of TNT biodegradation studies [41,42]. In comparison with other fungal species, *Trichoderma* species have shown a better ability to remove TNT, DNT, and their transient intermediates (amines) in pure cultures. These results suggest that fungi such as *Trichoderma* may have the potential to be used in biological decontamination systems, while further research studies should be directed toward TNT and/or DNT biodegradation capacities of these fungi [40,43]. Also, populations of soil micro fungi were shown to have a high natural variation of TNT tolerance and biotransformation ability, irrespective of any previous long-term exposure to this xenobiotic. The *Trichoderma* species was one of the mitosporic fungi that showed a higher tolerance and also had a high capacity to biotransform the compounds of TNT [40,43,44].

Based on the aforementioned facts relating to the ability of microorganisms, particularly *Trichoderma* species, to degrade and remediate toxicants and nitrogenous explosives [40,41,42,43,45], the current study was designed to evaluate the ability of *T. viride* to degrade nitrogenous TNT explosives by microbiological assays and GC/MS analysis. In this study, *T. viride* was raised on media containing TNT at doses of 50 and 100 ppm, respectively. The ability of *T. viride* to decompose TNT was evaluated in growth media using GC-MS analysis.”

## 2. Results and Discussion

In this study, *T. viride* fungus have the ability to decompose and use TNT explosives at doses of 50 and 100 ppm on its growth media as the nitrogenous source needed for normal growth. In addition, the GC/MS analysis confirmed the biodegradable efficiency of TNT, whereas the initial retention peak of TNT compounds disappeared, and another two peaks appeared at the retention times of 9.31 and 13.14 min.

### 2.1. Screening of Fungal Growth on TNT-Containing Media

**Figure 1:**
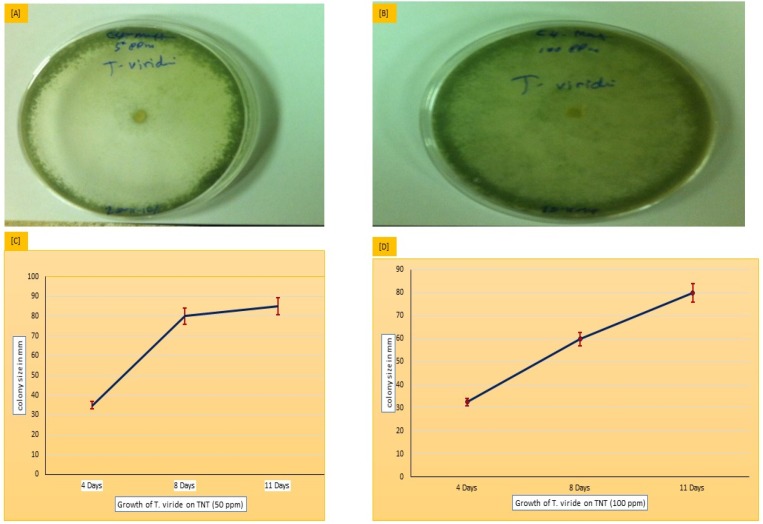
*T.viride* growth on malt agar suspended with TNT as a sole source of nitrogen at 50 ppm (**A**, **C**) and at 100 ppm (**B**, **D**). The growth of *T.viride* is indicated according to the size of the colony in mm.

**Figure 2:**
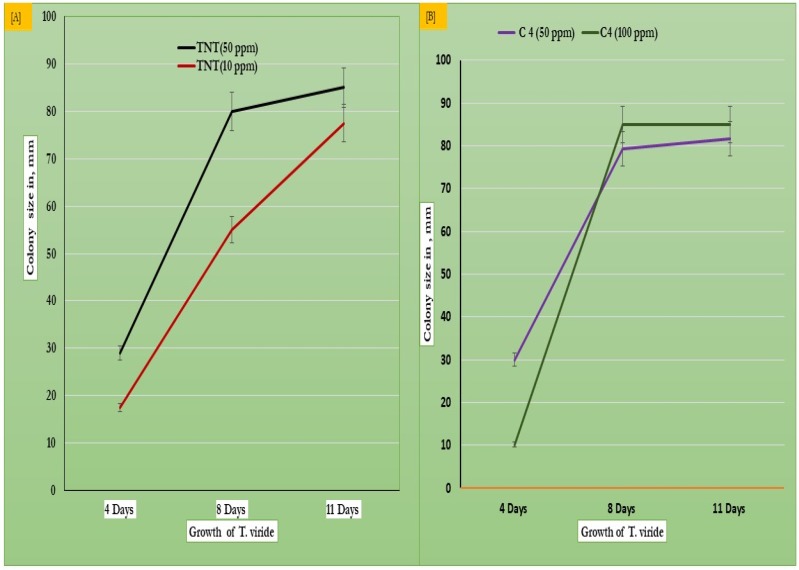
Differential growth of *T. viride* on dextrose agar (**A**) and malt agar media (**B**), treated with TNT at doses of 50 and 100 ppm respectively.

In order to measure the ability of the *T. viride* fungus to decompose and use TNT as a source for the nitrogen needed during growth, *T. viride* colonies were cultivated on malt extract agar medium and Sabouraud dextrose agar medium, as shown in Table 1 and Figure 1 and Figure 2. The experiment was repeated in triplicate, and the standard deviation (SD) was found to be below 2.1 for both concentrations.

As shown in Table 1, Figure 1a–d, and Figure 2, at TNT doses of 50 and 100 ppm, the results show that colony diameters significantly increase from 35.5 and 32.5 to record higher colony formation with a colony diameter of 85.0 mm at 8 and 11 days of growth. This confirms the biological use of TNT as a source of nitrogen, which is assimilated by T. viride on both the malt extract and Sabouraud dextrose agar medium (Figure 2a,b). The data obtained suggests that the fungus T. viride is significantly capable of decomposing TNT explosives, and uses them as a nitrogenous source for normal growth. It may be of interest to use this fungus as a good model in microbial-based bioremediation technologies against toxicological agents.

Previous studies showed that 2,4,6-TNT is a man-made substance that is released into the environment and represents a potential hazard due to toxicity, originating either from 2,4,6-TNT substances, or its transformed metabolites during the manufacturing process, or in the process of incomplete combustion [1]. These compounds have been shown to have a low biodegradability and higher persistence in the environment [2,3]. In addition, it was reported that the transformation products of TNT are teratogenic, cytotoxic, and may cause cell mutations in animal experimental models [17,18]. However, the carcinogenic effects of TNT on humans still need to be explored [17,18]. This may be related to the eco-toxicological effects and persistence of TNT and its transformation products in the environment, which significantly affect a wide range of ecological receptors, particularly microorganisms, algae, plants, invertebrates, some vertebrates, and humans [19,20,21].

It was reported that microbial-based bioremediation significantly uses the in situ microbial community to remediate toxic contaminates and return the polluted environment to its original state, or at least minimize the toxicity of the environment toward the normal range [28,29,30,31,32]. Previous research showed that bioremediation through transformation mechanisms has no or little benefit, and may lead to transformation into chemically unstable toxic organic compounds [46,47]. However, many fungal species have the ability to decompose xenobiotic alien vehicles, including nitrogenous explosives, by using certain cellular degrading enzymes [30,31,32].

### 2.2. Determination of TNT and Metabolites by GC/MS Analysis

**Figure 3:**
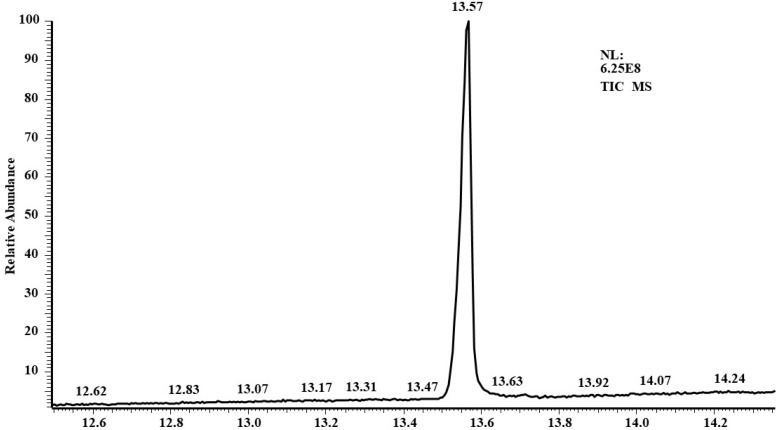
TNT sample determination by GC-MS. The chromatogram shows the peak of a TNT reference dissolved in acetone (1 mL acetone containing 50 ppm or 100 ppm TNT).

**Figure 4:**
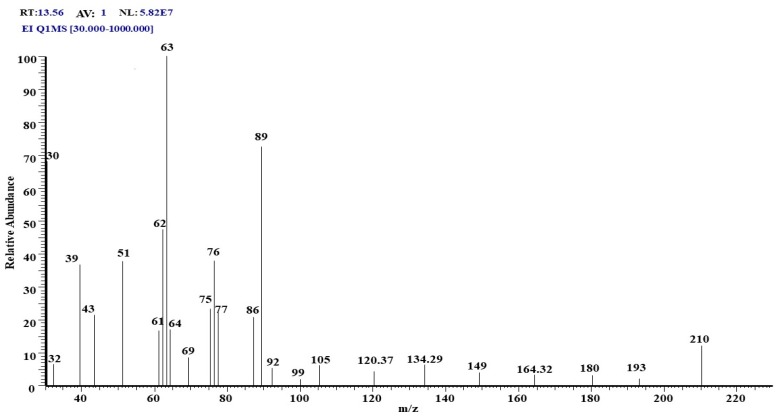
This spectrum corresponds to 2-methyl-1,3,5-trinitrobenzene (TNT), which has the following formula: C_7_H_5_N_3_O_6_, and a molecular weight of 227 g·mol^−1^.

To further elucidate the biological activity of *T. viride* to decompose TNT, GC/MS analysis was conducted to search the presence of TNT in growth media before and after growth at two doses (50 and 100 ppm). At the initial time of growth (zero time), GC/MS analysis showed a visible peak of 2-methyl-1,3,5-trinitrobenzene (TNT) on the chromatogram at the retention time of 13.57 min (Figure 3). The structure was evaluated by mass spectroscopy, and the results show a significant molecular structure formula (C_7_H_5_N_3_O_6_), with a molecular weight of 227 g·mol^−1^ (Figure 4). No other compounds are visible on the chromatogram except for some very small peaks, which are considered as impurities (Figure 4).

**Figure 5:**
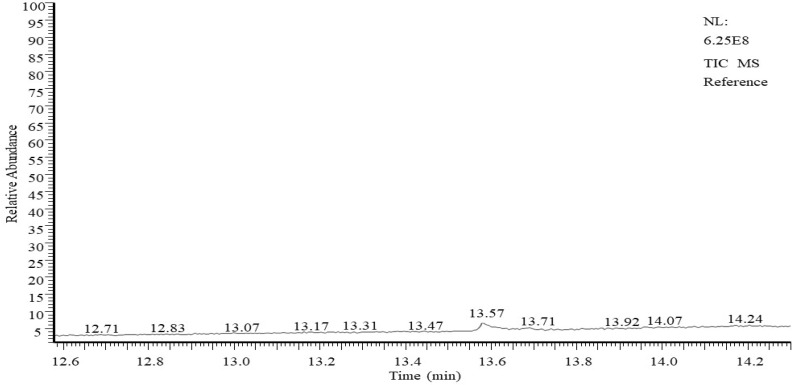
Chromatogram for 50 ppm TNT with a *T. viride* culture.

When TNT was used at doses of 50 and 100 ppm in the growth media of T. viride, the analyzed media solution following growth for 8–11 days, showed no detectable peaks for TNT on the chromatogram chart (Figure 5). This indicates that all TNT compounds were degraded in the medium by the biological decomposing action of T.viride at doses of 50 and 100 ppm.

**Figure 6:**
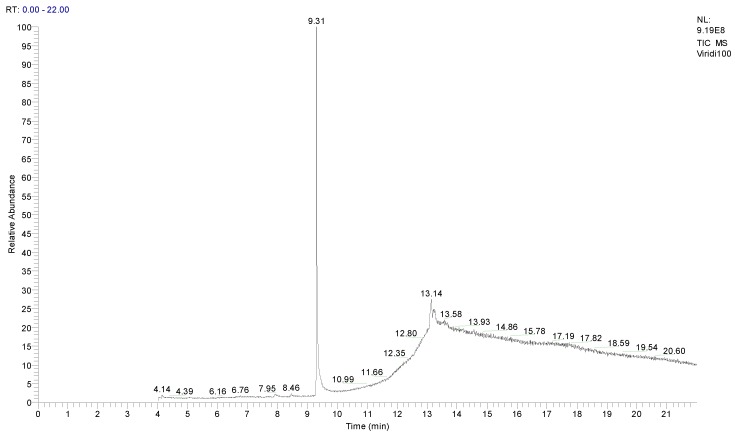
Chromatogram of 100 ppm TNT after treatment with *T. viride*.

**Figure 7:**
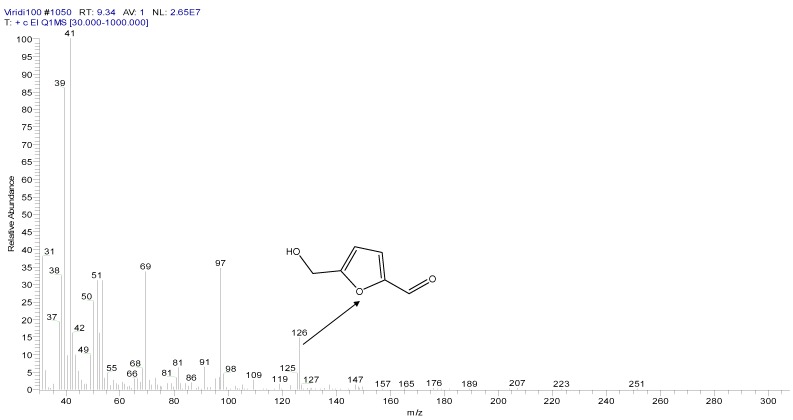
Mass spectrum of main peak, 5-(hydroxymethyl)-2-furancarboxaldehyde.

**Figure 8:**
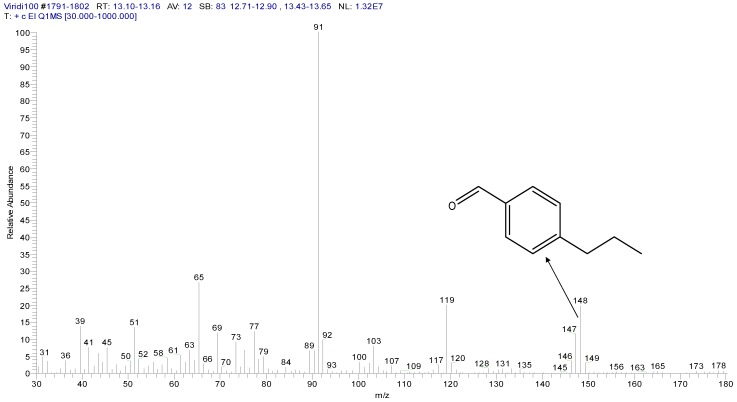
Mass spectrum of the minor component, 4-propyl benzaldehyde.

Figure 6 shows new peaks of the analyzed media following the growth of *T. viride.* Two main peaks can be observed: one peak appears at the retention time of 9.31 min, for a compound that was identified as 5-(hydroxymethyl)-2-furancarboxaldehyde with the molecular formula C_6_H_6_O_3_ and a molecular weight of 126 g·mol^−1^, as shown in Figure 7. As *T.viride* was grown on respective growth media, the detected compound C_6_H_6_O_3_ was shown to be the major degradation product of TNT. However, another peak was observed by GC/MS analysis at the retention time of 13.14 min, which was identified as 4-propyl benzaldehyde, with a formula of C_10_H_12_O and a molecular weight of 148 g·mol^−1^, as shown in Figure 8. The detected compound C_10_H_12_O is the minor degradation product of TNT decomposition, obtained under the conditions required for the growth of the *T. viride.*

In this study, as shown in Figure 5, Figure 6, Figure 7 and Figure 8, TNT was degraded by *T. viride* to other new compounds, as identified by the GC/MS analysis. The results showed that the initial peak corresponding to TNT compounds disappeared, and another two peaks appeared, which represent new compounds at the retention times of 9.31 and 13.14 min. Mass spectrum analysis identified 5-(hydroxymethyl)-2-furancarboxaldehyde with the molecular formula C_6_H_6_O_3_ and molecular weight of 126 g·mol^−1^ as the major compound, and 4-propyl benzaldehyde with the formula of C_10_H_12_O and a molecular weight of 148 g·mol^−1^ as a minor compound, both resulting from the biodegradation of TNT following the growth process of *T. viride* for 8–11 days. The formation of these possible degradation products was confirmed by matching their mass spectra with the library, which showed a probability of 90% for both products. In addition, in the chromatogram after TNT degradation, no peaks other than these two degradation compounds were noticed. This may confirm the degradation of TNT compound via cellular enzymatic action.

Even though TNT microbial degradation was studied over the years, the major challenge in this area is the resistance and refraction of these types of compounds to biological degradation, chemical oxidation, and hydrolysis. The resistance shown towards the biodegradation methods could be due to the symmetrical arrangement of three nitro-groups and a methyl group on the aromatic ring, coupled with strong electron-withdrawing properties of the nitro group, which limits the attack of the aryl group by dioxygenase enzymes. These structural arrangements prevent the aromatic ring with an electron shortage from acting as an electrophilic oxygenation mechanism, hindering its mineralization and removal from the contaminated sites [7,8,12,22,48]. In addition, the resistance of TNT to complete mineralization is also due to the easy reduction of nitro groups into amino groups, and the ultimate chemical misrouting reactions of its intermediates, in particular, triaminotoluene (TAT) [49,50].

**Scheme 1:**
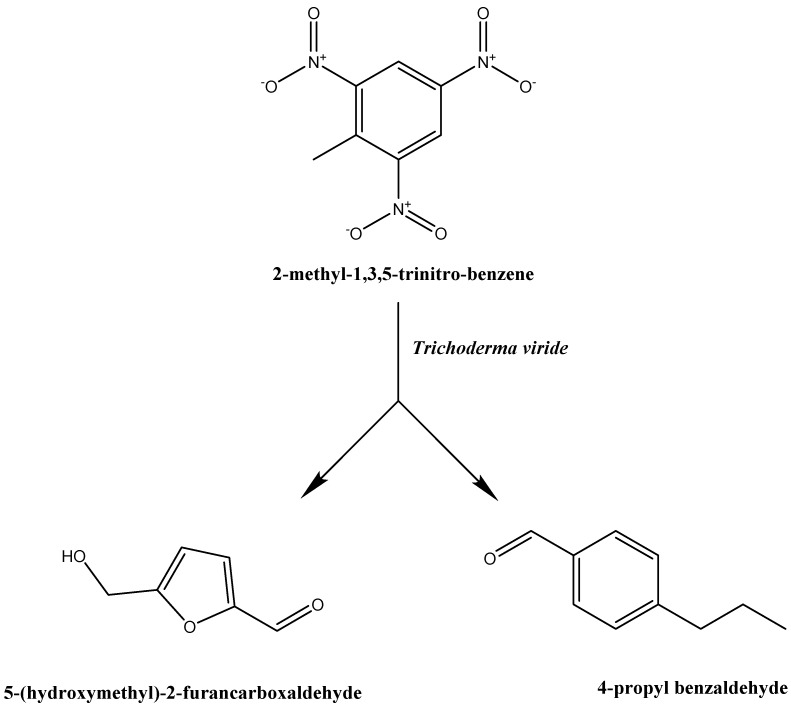
Postulated degradation of the 2-methyl-1,3,5-trinitrobenzene mechanism of *T. viride*. It is postulated that TNT biodegradation may occur via biotransformation enzyme mechanisms.

Furthermore, it was reported that the non-mineralization of TNT is a direct consequence of irreversible sorption of this explosive and its transformation products by soil [33]. In our study, the degradation of TNT compounds by *T. viride* may proceed via biotransformation mechanisms that facilitate the elimination of nitrogen and recyclization of the cleaved compounds. Thus, the GC/MS analysis supports and confirms that T.viride can use TNT as a sole source of nitrogen and has a cellular enzymatic degradable activity to bioremediate TNT compounds to other compounds, which are present in the growth media in major (C_6_H_6_O_3_) and minor (C_10_H_12_O) amounts, following the growth process for 8–11 days, as shown in Scheme 1. Previous studies showed that the resistance of TNT mineralization could be resolved by the activation of lignin peroxidase through the activation of the nitroreductase enzymes hydroxylaminodinitrotoluenes (HADNT) and aminodinitrotoluenes (ADNT) [51,52,53,54,55,56,57,58,59,60,61]. The latter enzymes are responsible for nitro group reduction, while the former catalyzes oxidation and aromatic ring cleavage [51,52,53].

Finally, microorganisms can be considered a biological tool for removing organic toxic substances because they are able to carry out biological activities to degrade, concentrate, remove, or even recover highly toxic chemical substances from contaminated environments [54]. In our study, T. viride was shown to have a potential biological activity to degrade TNT organic explosives. We postulate that TNT biodegradation may occur via biotransformation using certain degrading microbial enzymes. Thus, T. viride could be used as a target agent for microbial-based bioremediation technologies. Microorganisms have been used as an alternative strategy for conventional treatments against toxic organic substances and heavy metals [54,55,56,57].

## 3. Material and Methods

### 3.1. Chemicals and Biological Materials

#### 3.1.1. Chemicals and Preparations

TNT was obtained from the Criminal Investigation Department at the Ministry of Interior, Riyadh, Saudi Arabia. The purity of TNT was 95%. TNT/acetone stock solution was prepared by dissolving 48 mg of TNT in 50 mL of acetone to give a concentration of 960 ppm (mg·L^−^¹) of TNT. Malt agar and dextrose agar media were autoclaved for fifteen minutes, then allowed to cool and poured into Petri plates prior to the addition of TNT, and the whole plates were then ready for the screening of fungal growth.

#### 3.1.2. *T. viride* Specimens

Soil specimens were collected from an explosion area in Riyadh, Saudi Arabia for the isolation of *T. viride*. A serial dilution of each sample was prepared in sterilized distilled water. One milliliter of a diluted specimen was speared on the surface of *T. viride* selective medium (TSM) [8,58]. All plates were incubated at 25 ± 2 °C for 3 days. Colonies were purified in potato dextrose agar (PDA). The purified isolates were morphologically identified and stored at 4 °C and used during this study.

### 3.2. Screening of Fungal Growth on TNT Containing Media

Control cultures were screened on both malt agar medium and dextrose agar medium, which are designed to contain the proper formulation of carbon, protein, and nutrient sources essential for growth. Dextrose was added to the medium to provide a carbon and energy source for fungi. Additionally, malt extract agar contains digests of animal tissues (peptones), which provide a nutritious source of amino acids and nitrogenous compounds for the growth of fungi. In addition, acetone and Dimethyl Sulfoxide (DMSO) but no TNT were added to culture media (acetone and DMSO did not inhibit fungal growth).

In order to study the use of TNT as a source of nitrogenous compounds, the growth of *T. viride* was screened on both malt agar and dextrose agar growth media devoid of animal tissue (peptones), which provide a nutritious source of amino acids and nitrogenous compounds. The cultures were incubated in a shaker at 24 °C and 200 rpm. After two days of growth, each received 1 mL of acetone containing 50 or 100 ppm TNT and was amended with 25 µL of DMSO to help solubilize the TNT. Cultures were extracted three days after the addition of the TNT. TNT was added to the culture media either for three days before, or immediately before, extraction. To determine growth, replicate cultures were filtered, dried, and weighed.

### 3.3. Determination of TNT and Metabolites by GC/MS Analysis

Cultures of *T. viride* were homogenized in an explosion-proof blender with 50 mL of acetone. This was left to stand for 15 min, followed by filtration through filter paper [58,59]. Filtrates were extracted in a separating funnel with 100 mL of methylene chloride. Organic fractions evaporated overnight and were re-suspended in 5 mL of methylene chloride, and stored at −20 °C. At least four replicate cultures per fungus were analyzed. The extraction method used was designed to recover precipitated TNT, since TNT was added at 50 and 100 ppm [59].

A Thermo Scientific™ UltraFast TRACE GC with a Thermo electron-impact ionization (EI) TSQ Quantum™ Triple Quadrupole Mass Spectrometer (Waltham, MA, USA), equipped with a Phenomenex Zebron ZB-5MS (5 m × 0.25 mm i.d. × 0.25 µm film thickness or equivalent) column (411 Madrid Avenue, Torrance, CA, USA), was used in this protocol to separate and quantify TNT and its degradation products in the concentrated organic fractions. Organic culture extracts were analyzed by GC–MS, as previously reported [60,61], at different dilutions (10, 25, 50, 100, 250, 500, 1000, and 2000 ng/mL).

## 4. Conclusions

In this study, *T. viride* fungus showed a high natural variation in TNT tolerance, biodegradation, and biotransformation ability, and was able to use TNT explosives at doses of 50 and 100 ppm as a nitrogenous source for normal growth. In addition, the biodegradable efficiency of TNT explosives by *T.viride* was confirmed by using GC/MS analysis. Mass spectrum analysis identified 5-(hydroxymethyl)-2-furancarboxaldehyde with the molecular formula C_6_H_6_O_3_ and a molecular weight of 126 g·mol^−1^ (*m*/*z*) as the major compound, and 4-propyl benzaldehyde with a formula of C_10_H_12_O and a molecular weight of 148 g·mol^−1^ (*m*/*z*) as the minor compound, both resulting from the biodegradation of TNT following the growth process of *T. viride.*

Future molecular-based studies should be conducted to clearly identify the enzymes and corresponding genes responsible for the ability of *T. viride* to degrade and remediate TNT explosives. This could help in the eradication of soils contaminated with explosives or other toxic biohazards.

## Figures and Tables

**Table 1:** Presents the radial growth measurements of the studied fungi at 50 and 100 ppm 2,4,6-trinitrotoluene (TNT) as the sole nitrogen source with malt agar medium.

Fungi	After 4 Days				After 8 Days				After 11 Days			
	Control		TNT ± SD		Control		TNT ± SD		Control		TNT ± SD	
	C_1_	C_2_	50 ppm	100 ppm	C_1_	C_2_	50 ppm	100 ppm	C_1_	C_2_	50 ppm	100 ppm
*T. viride*	80.0	85.0	35.0 ± 2.0	32.5 ± 1.8	85.0	85.0	85.0 ± 1.9	85.0 ± 1.3	85.0	85.0	85.0 ± 2.1	85.0 ± 2.0

The growth of *T. viride* expressed by diameter of the mean colony (mm). C1 = Control 1 (malt extract agar medium) and C2 = Control 2 (sabouraud dextrose agar medium).
